# Calmodulin-dependent and calmodulin-independent glutamate decarboxylases in apple fruit

**DOI:** 10.1186/1471-2229-13-144

**Published:** 2013-09-28

**Authors:** Christopher P Trobacher, Adel Zarei, Jingyun Liu, Shawn M Clark, Gale G Bozzo, Barry J Shelp

**Affiliations:** 1 Department of Plant Agriculture, University of Guelph, Guelph, ON N1G 2W1, Canada

**Keywords:** Abiotic stress, Apple fruit, Biochemical regulation, Calmodulin, Controlled atmosphere storage, γ-Aminobutyrate, Glutamate decarboxylase, Recombinant protein

## Abstract

**Background:**

The ubiquitous, non-proteinaceous amino acid GABA (γ-aminobutyrate) accumulates in plants subjected to abiotic stresses such as chilling, O_2_ deficiency and elevated CO_2_. Recent evidence indicates that controlled atmosphere storage causes the accumulation of GABA in apple (*Malus* x *domestica* Borkh.) fruit, and now there is increasing interest in the biochemical mechanisms responsible for this phenomenon. Here, we investigated whether this phenomenon could be mediated via Ca^2+^/calmodulin (CaM) activation of glutamate decarboxylase (GAD) activity.

**Results:**

GAD activity in cell-free extracts of apple fruit was stimulated by Ca^2+^/CaM at physiological pH, but not at the acidic pH optimum. Based on bioinformatics analysis of the apple genome, three apple *GAD* genes were identified and their expression determined in various apple organs, including fruit. Like recombinant *Arabidopsis* GAD1, the activity and spectral properties of recombinant *Md*GAD1 and *Md*GAD2 were regulated by Ca^2+^/CaM at physiological pH and both enzymes possessed a highly conserved CaM-binding domain that was autoinhibitory. In contrast, the activity and spectral properties of recombinant *Md*GAD3 were not affected by Ca^2+^/CaM and they were much less sensitive to pH than *Md*GAD1, *Md*GAD2 and *Arabidopsis* GAD1; furthermore, the C-terminal region neither bound CaM nor functioned as an autoinhibitory domain.

**Conclusions:**

Plant GADs typically differ from microbial and animal GAD enzymes in possessing a C-terminal 30–50 amino acid residue CaM-binding domain. To date, rice GAD2 is the only exception to this generalization; notably, the C-terminal region of this enzyme still functions as an autoinhibitory domain. In the present study, apple fruit were found to contain two CaM-dependent GADs, as well as a novel CaM-independent GAD that does not possess a C-terminal autoinhibitory domain.

## Background

The accumulation of γ-aminobutyrate (GABA) in plants subjected to abiotic stress is often attributed to the Ca^2+^/calmodulin (CaM)- or pH-mediated stimulation of glutamate decarboxylation, although polyamines may also contribute [1-3]. Plant glutamate decarboxylase (GAD) requires pyridoxal 5′-phosphate (PLP) as a co-factor, is specific for L-glutamate and maximally active at approximately pH 5.8, and exists as dimeric or hexameric complexes [1]. GAD activity in cell-free extracts prepared in the presence of protease inhibitors and partially purified by ammonium sulfate precipitation and ion-exchange chromatography is typically assayed as the production of ^14^CO_2_ from radiolabeled glutamate or GABA from unlabeled glutamate with or without the addition of CaM antagonists such as trifluoperazine (TFP). The activation of GAD activity by Ca^2+^/CaM is more dramatic at neutral pH than optimum pH.

The C-terminal CaM-binding domain (CaMBD) of GAD is highly variable and evidence exists for a C-terminal domain in rice GAD (*Os*GAD2) that does not bind CaM, but is autoinhibitory [1,4,5]. The extent of the Ca^2+^/CaM stimulation of activity can also vary widely among various recombinant GADs (5- to 60-fold) [6-10]. Possible explanations for this variability could be the final purity of the protein being tested, as well as the method used for purification. There is also evidence to suggest that a tag may influence yield, conformation and biological activity of the recombinant protein [11]; however, the time required to remove the motif may be detrimental to the activity of an unstable protein.

The accumulation of GABA in apple fruit stored under controlled atmosphere conditions was recently reported [12-14], and now there is increasing interest in the biochemical mechanisms responsible for this phenomenon [3,14]. Here, we report the identification of three GADs in apple fruit and demonstrate, via the use of suitable tagging/purification strategies, that two are CaM-dependent and one is CaM-independent.

## Results

### GAD activity in cell-free extracts from apple fruit

GAD activity in cell-free apple extracts at pH 5.5 in the absence or presence of Ca^2+^/CaM was similar with bis(2-hydroxyethyl)amino-tris(hydroxymethyl)methane (Bis-Tris–HCl) and pyridine-HCl buffers (data not shown). Thus, we used these two buffers to study GAD activity at several pHs between 5.0 and 7.0 (Table 1). The pH optimum was approximately 5.5 to 6.0, and at these pHs Ca^2+^/CaM stimulated activity by about 70% and TFP inhibited activity by 35-60% in the presence of Ca^2+^/CaM. At pH 6.5 and pH 7, the stimulation by Ca^2+^/CaM (440-670%) and the inhibition by TFP increased markedly. TFP did not affect activity in the absence of added Ca^2+^/CaM.

**Table 1:** **Effect of pH, Ca**^**2+**^**/CaM and TFP on GAD activity in cell-free apple extracts**

**Treatment**	**pH**				
	**5.0**	**5.5**	**6.0**	**6.5**	**7.0**
	nmol mg prot^-1^ min^-1^				
+Ca^2+^/CaM -TFP	7.48±0.58a^1^	14.69±2.92a	12.91±0.74a	3.66±1.18a	0.54±0.21a
+Ca^2+^/CaM + TFP	4.81±0.97b	9.21±1.36a	8.59±1.56b	0.86±0.09b	0.09±0.02b
-Ca^2+^/CaM -TFP	3.73±0.56b	9.91±2.21a	8.63±0.33b	1.03±0.09b	0.11±0.06b
-Ca^2+^/CaM + TFP	4.62±0.65b	8.76±1.30a	7.39±1.23b	0.68±0.09b	0.07±0.01b
% Ca^2+^**/**CaM stimulation^2^	62	68	75	440	670

^1^Data represent the mean ± SE of three independent preparations at each pH; data in a column and sharing the same letter are not significantly different according to Tukey’s test (P > 0.05).

^2^Calculated from activities at + Ca^2+^/CaM –TFP and -Ca^2+^/CaM + TFP.

### Alignment of three putative apple GADs with previously characterized plant GADs

**Figure 1:**
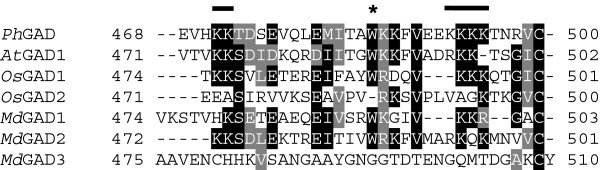
**Multiple sequence alignment of the C-terminal segments of plant GADs.** Multiple sequence alignment was created with Clustal W [15] and edited manually. The C-terminal CaMBDs of *Petunia hybrida* (*Ph*GAD) [16], *Arabidopsis thaliana* (*At*GAD1) [18] and *Oryza sativa* (*Os*GAD1) [5] have been characterized experimentally. Two conserved positively charged clusters of lysines that flank the CaMBD are marked with black lines [9]. The conserved tryptophan residue involved in CaM binding [16,17], marked with an asterisk, is not present in *Os*GAD2, which does not bind CaM [5]. Rice GAD numbering is taken from Akama and Takaiwa [5]: *Os*GAD1 (AB056060, LOC_Os08g36320.1); *Os*GAD2 (AB056061, LOC_Os04g37500.1). Identical residues are shown with a black background, and similar residues are shown with a grey background. All enzymes were identified as belonging to the aspartate aminotransferase superfamily (fold type I) of PLP-dependent enzymes by the NCBI CD-Search tool [18].

*MdGAD1* (GenBank Acc. No. KC812242), *MdGAD2* (GenBank Acc. No. KC812243) and *MdGAD3* (GenBank Acc. No. KC812244), respectively, encode proteins of 503 (56.6 kDa), 501 (56.9 kDa) and 510 (57.0 kDa) amino acids, which are 64-75% identical to each other and 60-85% identical to several plant GADs that have been characterized experimentally (see the multiple sequence alignment, based on Clustal W [15], of *Ph*GAD, *At*GAD1, *Os*GAD1-2, and *Md*GAD1-3 in Additional file 1: Figure S1). The region of greatest variability in the sequences of these proteins is in the C-terminus, the location of the CaMBD [9,16,17]. The C-terminal segments of the three apple GADs are 3-27% identical to each other and 4-46% identical to those in GADs of the other plant species, although this region of *Md*GAD3 has only 3-6% identity. Closer examination and manual editing of the sequence alignment indicates that two conserved positively charged clusters of lysines flanking the CaMBD and a conserved tryptophan residue are involved in CaM binding [9,18] (Figure 1). Notably*, Os*GAD2 is missing the first lysine cluster and the tryptophan and does not bind CaM [5]. In general, both *Md*GAD1 and *Md*GAD2 contain most of the conserved features, but *Md*GAD3 contains none of these features.

### Expression of three putative apple GAD genes in various plant organs

**Figure 2:**
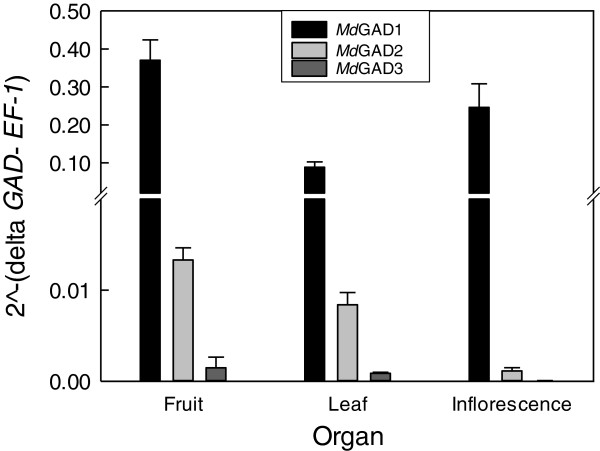
**Relative expression of three apple *GAD*s in various plant organs.** While *MdGAD3* was detectable, with the scale chosen for presentation, the low expression level is not evident. Data represent the mean ± SE of three biological replicates.

Transcripts for the three GAD genes were detectable in the fruit, leaf and inflorescence, but *Md*GAD1 was the most prominent, followed distantly by *Md*GAD2 and *Md*GAD3 (Figure 2). The relative proportions of *Md*GAD2 and *Md*GAD3, compared to *Md*GAD1, were highest in the fruit and lowest in the inflorescence.

### Impact of pH and Ca^2+^/CaM on the activity of various recombinant apple and *Arabidopsis* GADs

We investigated the impact of Ca^2+^/CaM on the performance (i.e., activity and spectral properties at various pHs) of recombinant full-length *Md*GAD1-3 and truncated *Md*GAD3ΔC34. Apple GAD activity was compared to the performance of the full-length *At*GAD1 and in most cases a truncated version of the *At*GAD1 lacking the C-terminal CaMBD (*At*GAD1ΔC32), ensuring that the purification and assay of the enzymes were effective. Because *At*GAD1ΔC32, *Md*GAD3 and *Md*GADΔC34 could not be purified by CaM-affinity chromatography, a 6-His motif was added (designated by the prefix H on the protein name), prompting us to also investigate the impact of the tag on GAD activity. Assays of GAD activity and determination of spectral properties were simultaneously conducted as soon as possible after the recombinant protein was extracted to minimize proteolysis that seemed to occur even in the presence of several popular protease inhibitors.

**Figure 3:**
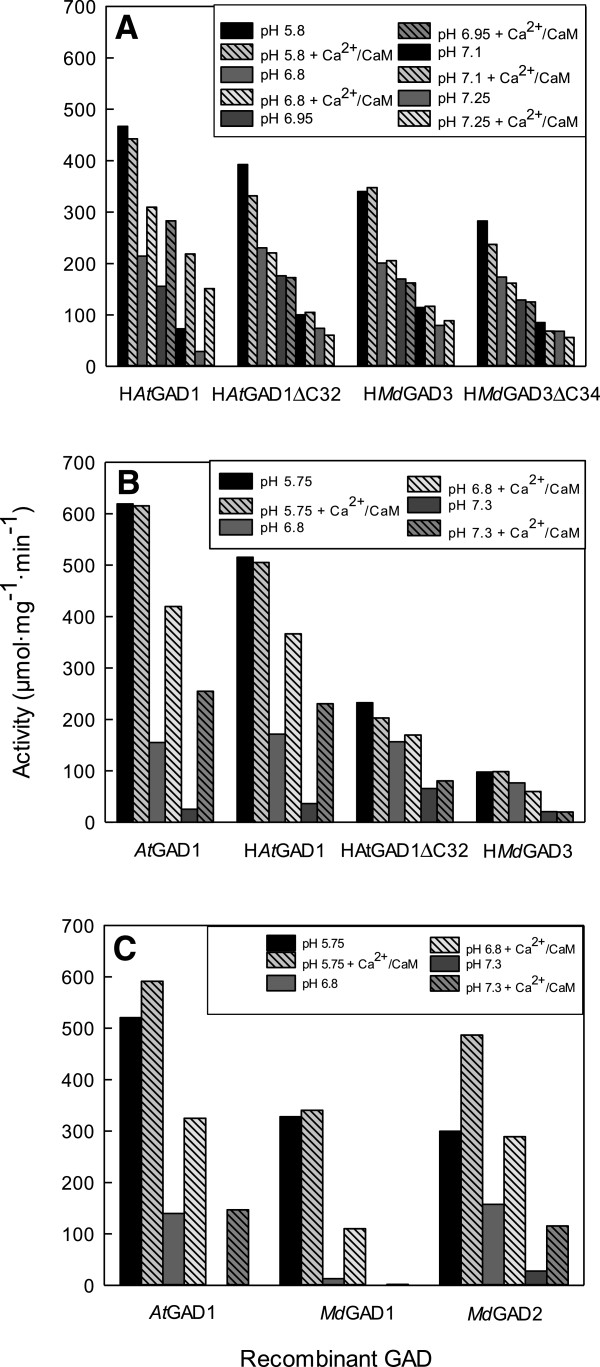
**Impact of pH and Ca**^**2+**^**/calmodulin on the activity of various recombinant apple and *Arabidopsis *GADs.** Each panel **(A-C)** represents a separate experiment and shows results from recombinant proteins prepared simultaneously. The data represent the mean of three technical replicates for each treatment. All proteins, except those tagged with a 6-His motif (designated with the prefix H), were purified by calmodulin-affinity chromatography. While the activity of *Md*GAD1 was detectable at pH 7.3, with the scale chosen for presentation, the low activity is not evident in the figure. SDS-PAGE analysis of the expression and purification of these proteins is given in Additional file 2: Figure S2.

Initially, the N-terminal His-tagged versions of *Md*GAD3, *Md*GAD3ΔC34, *At*GAD1, and *At*GAD1ΔC32 were compared. After purification via Ni^2+^- affinity chromatography, these recombinant proteins were thrombin-digested to remove the His-tag and concentrated for determining activity and spectral properties. Each of these preparations had a prominent band on a SDS-PAGE gel at the predicted molecular mass; only the preparation of H*At*GAD1 appeared to contain a second prominent band, which was of a slightly lower molecular mass than the other and possibly a degradation product resulting from the sensitivity of the CAMBD to proteolytic activity (Additional file 2: Figure S2). The highest activities of the various GADs were evident at pH 5.8 and varied less than 1-fold (approximately 280 to 470 μmol mg prot^-1^ min^-1^), and there was no stimulation of these activities by added Ca^2+^/CaM (Figure 3A). Activities declined with increasing pH and for H*At*GAD1 this was accompanied by 2- and 4-fold stimulations by Ca^2+^/CaM of activity at pHs 7.1 and 7.25, respectively. However, there was no stimulation of H*At*GAD1ΔC32, H*Md*GAD3 and H*Md*GAD3ΔC34 activities by Ca^2+^/CaM at these pHs. Since both H*At*GAD1 and H*At*GAD1ΔC32 responded to Ca^2+^/CaM as predicted, these data support the notion that Ca^2+^/CaM could not bind to either H*Md*GAD3 or H*Md*GAD3ΔC34.

In a follow-up experiment, H*Md*GAD3 was compared to H*At*GAD1, H*At*GAD1ΔC32 and *At*GAD1, with the first three recombinant proteins being purified by Ni^2+^-affinity chromatography and the fourth by CaM-affinity chromatography. Steps involving thrombin digestion and concentration were omitted. As above, preparations of purified H*At*GAD1, as well as *At*GAD1, had one main band at the predicted molecular mass on a SDS-PAGE gel, as well as a slightly smaller prominent band; the other GADs showed only one main band (Additional file 2: Figure S2). Activities reached 620 μmol mg prot^-1^ min^-1^ at pH 5, decreased with increasing pH, and appeared to be more variable than in the initial experiment (Figure 3B). Ca^2+^/CaM stimulated the activities of *At*GAD1 and H*At*GAD1 at pH 7.3 by 9- and 5-fold, respectively, but had no impact on the activities of H*At*GADΔC32 and H*Md*GAD3. Therefore, the response of H*At*GAD1, H*At*GADΔC32 and H*Md*GAD3 to Ca^2+^/CaM was similar to that in the initial experiment. Notably, the impact of Ca^2+^/CaM was slightly greater with the *At*GAD1, suggesting that the presence of the His tag influences the conformation of the protein. Overall, these results confirm that the C-terminus of *At*GAD1 is autoinhibitory at neutral pH and that this autoinhibition can be relieved when bound to Ca^2+^/CaM. They also indicate that the C-terminus of *Md*GAD3 is not autoinhibitory and that it does not bind Ca^2+^/CaM.

Subsequently, *Md*GAD1 and *Md*GAD2 were compared to *At*GAD1. All three recombinant proteins were purified by CaM-affinity chromatography and steps involving thrombin digestion and concentration were omitted (Figure 3C). Purified *At*GAD1 had one prominent and one minor band, *Md*GAD1 had one major band, and *Md*GAD2 had two bands of equal prominence (Additional file 2: Figure S2). The higher molecular mass bands were equivalent to the predicted subunit molecular masses for the three proteins. The activity of *At*GAD1 at pH 5 was similar to that in the previous experiment, but in this case the stimulation by Ca^2+^/CaM at pH 7.3 was 150-fold because the activity in the absence of Ca^2+^/CaM was very low (Figure 3C). The activities of *Md*GAD1 and *Md*GAD2 at pH 7.3 and 6.8, respectively, were stimulated by approximately 1- and 9-fold, and 3- and 1-fold. These results indicate that *Md*GAD1 and *Md*GAD2, like *At*GAD1, have an autoinhibitory domain, but the pH optimum for *Md*GAD1 and *Md*GAD2 may differ somewhat from each other.

### Impact of pH and Ca^2+^/CaM on the spectral properties of various recombinant apple and *Arabidopsis* GADs

**Figure 4:**
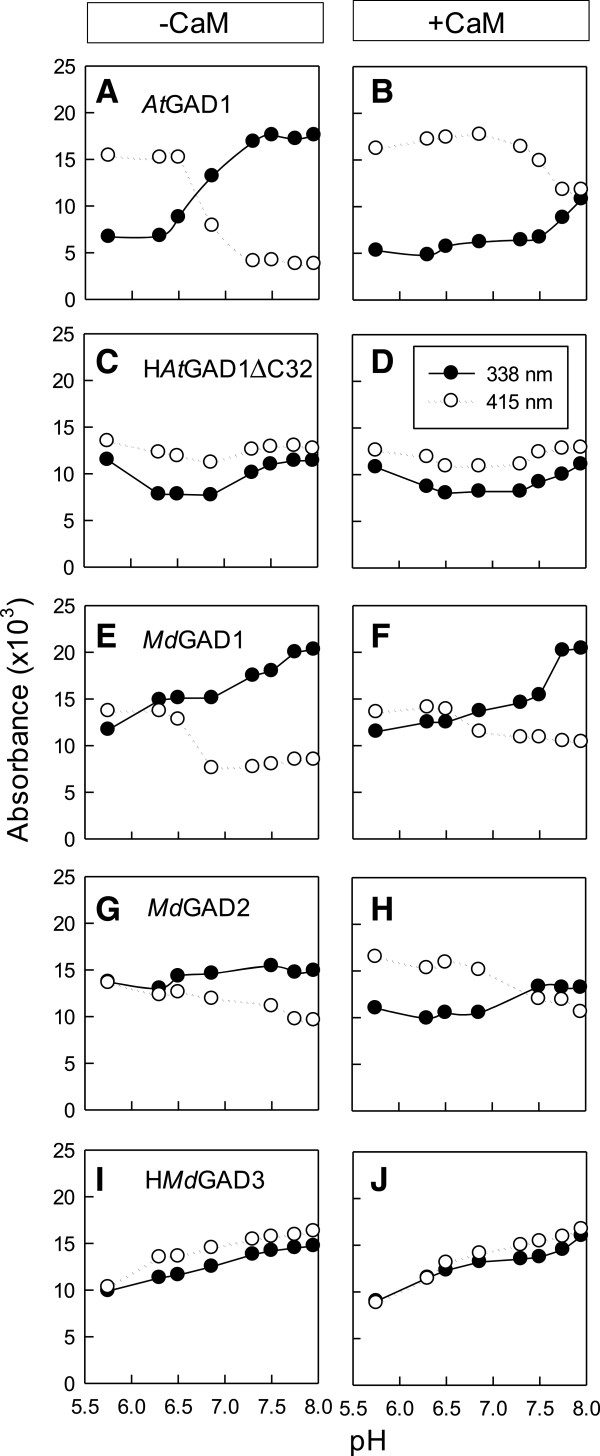
**Impact of pH and Ca**^**2+**^**/calmodulin on the spectral properties of various recombinant apple and *Arabidopsis *GADs.** These properties were determined on most of the purified proteins studied in Figure 3. The prefix H on some protein designations represents the 6-His motif. Absorbance at 338 nm and 415 nm, respectively, represents the enolimine and and ketoenamine forms of the internal aldimine. Each row of panels represents a different protein in the absence **(A, C, E, G, I)** and presence **(B, D, F, H, J)** of CaM.

Recently, Gut *et al*. [9] attributed absorption bands for *At*GAD1 at 338 nm and 415 nm, respectively, to the enolimine and ketoenamine tautomers of the Schiff base of PLP and enzyme (internal aldimine) (Additional file 1: Figure S1). Here, the absorption bands were compared among *At*GAD1, H*At*GAD1ΔC32, *Md*GAD1, MdGAD2 and H*Md*GAD3 (Figure 4). As found previously for *At*GAD1 [9], both bands were evident over the pH range of 4.75 to 7.95, the 415 nm band prevailed at low pH where the enzyme is most active and the 338 nm band prevailed at neutral and basic pHs, the absorption values at 338 nm or 415 nm displayed a sigmoidal curve with pH, and Ca^2+^/CaM essentially abolished the pH-dependence (Figure 4A and B). Also, the absorption bands for a truncated version of the protein lacking the CaMBD displayed a pH-independent pattern and was characterized by the prevalence of the 415 nm band over the entire pH range, regardless of whether Ca^2+^/CaM was present or not (Figure 4C and D); these characteristics are similar to those observed with a version of *At*GAD1 in which Lys496 and Lys 497 in the CaMBD are replaced by alanine residues [9] (Figure 1). Here, the prevalence of the bands was similar in both *Md*GAD1 and *Md*GAD2 at low pH, but the 415 nm band decreased with increasing pH and the 338 nm band increased, although the change in levels was not as dramatic as with *At*GAD1 and the sigmoidal relationship between absorbance and pH was less evident (Figure 4E and G). Nevertheless, Ca^2+^/CaM essentially abolished the pH dependence (Figure 4F and H). For H*Md*GAD3, the levels of the bands were similar, although they increased slightly over the pH range; Ca^2+^/CaM had no effect on this pattern (Figure 4I and J). Together, these data indicate that pH and Ca^2+^/CaM influence the spectral properties of *Md*GAD1 and *Md*GAD2 in a manner similar to that observed for *At*GAD1, although the magnitude of the response is less dramatic, and that H*Md*GAD3, like H*At*GAD1ΔC32, is essentially unaffected by pH and Ca^2+^/CaM.

## Discussion

### GAD activity in cell-free extracts of apple fruit was regulated by both pH and Ca^2+^/CaM

GAD enzymes are ubiquitous across all kingdoms, and in plants both transcript accumulation and activity may be induced during development and in response to abiotic and biotic stress perturbations [2,4]. In plants subjected to temperature stress, drought, salinity or mechanical handling, there is an increase in intracellular Ca^2+^ concentration [19], which would promote stimulation of GAD by Ca^2+^/CaM, whereas exposure to elevated CO_2_, O_2_ deficiency and wounding would influence GAD activity by cytosolic acidification in numerous plant tissues and species [19,20]. As GABA has been shown to accumulate in apple fruit during controlled atmosphere storage, and this level is higher during prolonged treatment with elevated CO_2_[12-14], we hypothesized that apple fruit GAD activity is stimulated by cytosolic acidification and/or Ca^2+^/CaM. GAD activity of cell-free extracts of apple fruit displayed optimal activity at pH 5.5 in the presence of PLP and saturating glutamate (Table 1); the final specific activity is comparable to *in vitro* activities of vegetative tissues of petunia, soybean, *Arabidopsis* and Maritime pine seedlings [1]. Apple fruit GAD activity was more dramatically stimulated by Ca^2+^/CaM at near physiological pH than at acidic pH (Table 1), indicating that it may be regulated *in planta* by both Ca^2+^/CaM and pH. As there was no effect of the CaM anatagonist, TFP, on *in vitro* GAD activity, it is suggested that GAD was not bound with endogenous CaM and the binding of Ca^2+^/CaM to GAD is tightly controlled by specific controlled atmosphere stress parameters. Overall, the biochemical properties of GAD activity in cell-free extracts of apple fruit provide support for the existence of a Ca^2+^/CaM-regulated GAD, as is typical of most plants [1]. Notably, three homologous *GAD* genes (Additional file 1: Figure S1), designated as *MdGAD1, MdGAD2* and *MdGAD3*, were present in the apple plant (Figure 2). *MdGAD1*, like *AtGAD2*, was abundant and ubiquitous, whereas *MdGAD2* and *MdGAD3*, like *AtGAD3* and *AtGAD4,* were expressed at much lower levels and might be candidates for induction in fruit by the stresses (i.e., chilling, O_2_ deficiency and elevated CO_2_) imposed during controlled atmosphere storage (Figure 2) [2,3].

### Recombinant GADs from apple fruit were both CaM-dependent and CaM-independent

With our tagging/purification strategies, we were able to confirm that recombinant *At*GAD1 is activated at physiological pH by Ca^2+^/CaM whether or not the 6-His tag was retained during the assay of activity and spectral properties (Figures 3 and 4). However, the degree of activation appeared to be greatest with the recombinant form of the native protein, which could be attributed to the very low activity found in the absence of Ca^2+^/CaM. While this interpretation is complicated somewhat by the variable amount of a smaller contaminating protein, probably a degradation product, in the final protein preparation, these findings do suggest that interpretation of the impact of Ca^2+^/CaM on the conformation and biological activity of tagged recombinant plant GADs should be done with caution [11].

Here, His-tagged or untagged versions of the three apple GADs were expressed in *Escherichia coli* and purified to homogeneity or near homogeneity (Additional file 2: Figure S2). The predicted subunit *M*_*r*_ of the three recombinant apple proteins (56.6-57 kDa) is similar to the majority of plant GADs, which exist as hexamers of 43–62 kDa subunits [1]. The recombinant apple GADs displayed a similar pH profile as shown for GAD activity in the cell-free extracts of apple fruit (Table 1, Figure 3), as well as a similar pH optimum with the majority of plant GADs, and activities with Ca^2+^/CaM at physiological pH that are as high or higher than their maximal activities in the literature [1]. Unlike most plant GADs, *Md*GAD3 was not activated by Ca^2+^/CaM at near physiological pHs, even though the technique/assay used in this study was clearly able to demonstrate such a relationship (Table 1, Figures 3 and 4). Historically, the binding of CaM to the conserved C-terminal domain has been considered necessary to relieve autoinhibition of GAD at physiological pH [1]. However, *Os*GAD2 (Figure 1) was found to be CaM-independent and possess a C-terminal region that is autoinhibitory [5]. *Md*GAD3 is only the second reported plant GAD that cannot be purified by CaM-affinity chromatography and whose activity and spectral properties are not affected by Ca^2+^/CaM. Notably, *Md*GAD3 is the first plant GAD that possesses a C-terminal region that is not autoinhibitory (Figure 3).

Alignment of the C-terminal region of *Md*GAD1 and *Md*GAD2 with biochemically characterized plant GADs demonstrated that key CaMBD residues are conserved in the apple enzymes, and that these are absent in *Os*GAD2 (Figure 1). The well-conserved tryptophan and a pair of lysine residues at the C-terminal end are crucial for interactions with CaM and the formation of high molecular weight oligomers in *Ph*GAD and *At*GAD1 [9,16,21,22]. In comparison to plant GADs known to be stimulated by Ca^2+^/CaM, *Md*GAD3 is missing two positively charged flanking regions (Lys474-Lys475 and Lys496-Lys497) and the Trp488-Lys489-Lys490-Phe491-Tyr492 motif (Figure 1). Their substitution by non-conserved residues may explain the lack of Ca^2+^/CaM stimulation of the recombinant *Md*GAD3. Interestingly, almost all CaM-binding residues in plant GADs are conserved in *Md*GAD1 and *Md*GAD2; the main exceptions in which charge is not conserved are substitutions at Lys474 and Lys 490 in *Md*GAD1 and Lys497 in *Md*GAD2 (Figure 1).

## Conclusions

Plant GADs typically differ from microbial and animal GAD enzymes in possessing a C-terminal 30–50 amino acid residue CaMBD [1,4,9]. To date, *Os*GAD2 is the only exception to this generalization; notably, the C-terminal region of this protein still functions as an autoinhibitory domain (5). In the present study, GAD activity in cell-free extracts of apple fruit could be stimulated by Ca^2+^/CaM at physiological pH. Based on bioinformatics analysis of the apple genome, we identified three apple *GAD* genes and then monitored their expression in various plant organs, including apple fruit. Like most plant GADs, the activity and spectral properties of recombinant *Md*GAD1 and *Md*GAD2 were regulated by both Ca^2+^/CaM and acidic pH and possessed a highly conserved CaMBD. In contrast, the activity and spectral properties of recombinant *Md*GAD3 were not affected by Ca^2+^/CaM and they were less sensitive to pH; furthermore, the C-terminal region neither bound CaM nor functioned as an autoinhibitory domain. These observations suggest that: (i) *Md*GAD3 is constitutively active, whereas *Os*GAD2 needs an activator other than Ca^2+^/CaM to be activated; and (ii) CaM–dependent and -independent *Md*GADs enzymes serve different roles in GABA production during the onset of physiological injury associated with controlled atmosphere storage conditions (12–14).

## Methods

### Preparation of cell-free extracts of apple fruit

'Empire’ apple (*Malus* × *domestica* Borkh.) fruit were purchased from a local supermarket and cell-free extracts were prepared essentially as described elsewhere [14,23]. A 10 to 60% (NH_4_)_2_SO_4_ cut was made and the resulting solution was desalted using an Econo-Pac 10DG column (Bio-Rad Laboratories) equilibrated with 100 mM Bis-Tris–HCl, pH 7.0, and 10% glycerol, and immediately assayed for GAD activity.

### Identification and cloning of GADs from apple and *Arabidopsis*

The 502 amino acid (aa) *Arabidopsis* GAD1 (At5g17330) sequence was used to query the apple expressed sequence tag database at the National Center for Biotechnology Information [24]. Alignment of multiple sequences produced a single candidate that would produce a 503 aa product. This sequence was in turn used to query predicted peptides from the apple genome [25]. Five significant hits were obtained: MDP0000284588 on chromosome 1; MDP0000307719 on chromosome 16; and, MDP0000587459, MDP0000322533 and MDP0000388356 on chromosome 9. Closer inspection of MDP0000322533 and MDP0000388356, and their alternative gene sets MDP0000234887 and MDP0000201843, respectively, revealed that these two regions on chromosome 9 have identical nucleotide sequences. Several attempts were made to clone these open reading frames (ORFs) from fruit and leaf cDNA samples, but none were successful.

MDP0000284588 had five more exons than the candidate sequence obtained from the EST data so two sets of primers based on both sequences were designed to amplify the corresponding cDNA. Primers GAD1-FP and GAD1-RP, designed to amplify the sequence identified via EST data, produced the expected 1506 bp product from fruit cDNA, designated *MdGAD1* (Additional file 1: Table S1 for all primer sequences used in this study). The primers CT-F32 and GAD1-RP, designed to amplify the longer version corresponding to MDP0000284588, did not produce a product from fruit or leaf cDNA samples. Comparing the *MdGAD1* sequence to the apple genome permitted identification of the 5′ and 3′ untranslated regions (UTRs); primers (GAD1-FP and GAD1-RP) in these regions were used to amplify the entire ORF plus portions of the UTRs.

Primers CT-F33 and CT-R33, designed to amplify MDP0000587549, produced the expected 1506 bp product designated *MdGAD2*. 5′- and 3′-Rapid Amplification of cDNA Ends (RACE) were used to confirm that *MdGAD2* was a full-length ORF. The SMART-RACE kit (Clontech, CA, USA) was used to prepare cDNA from total RNA according to the manufacturer’s instructions. The gene-specific primer CT-R37 was used for 5′-RACE with *MdGAD2*, and the primer CT-R38 was used for nested polymerase chain reaction (PCR). The gene-specific primer CT-F37 was used for 3′-RACE, and the primer CT-F38 was used for nested PCR.

Primers CT-F34 and CT-R34, designed to amplify MDP0000307719, produced the expected 1533 bp product from a leaf cDNA sample only; it was designated *MdGAD3*. Comparing the *MdGAD3* sequence to the apple genome permitted identification of the 5′ and 3′ UTRs; primers (CT-F44 and CT-R44) in these regions were used to amplify the entire ORF plus portions of the UTRs.

The *AtGAD1* ORF was amplified with 5′ *Nde*I and 3′ *BamH*I restriction sites with primers *At*GAD1-FP and *At*GAD1-RP and sub-cloned into pET15b (Invitrogen, USA) to give pET15b-*AtGAD1*. The *AtGAD1* ORF minus the 32 C-terminal residues that make up the CaMBD was amplified with 5′ *Nde*I and 3′ *BamH*I restriction sites with primers *At*GAD1-FP and *At*GAD1ΔCaMBD-RP; the resulting product was sub-cloned into pET15b to give pET15b-*AtGAD1*ΔCaMBD.

Two of the apple GADs were sub-cloned into pET15b after 5′ *Nde*I and 3′ *BamH*I restriction sites were added via PCR using primers aGL and aGR for *MdGAD1*, and primers CT-F39 and CT-R39 for *MdGAD2*. *MdGAD3* was also cloned into pET15b after addition of *Xho*I restriction sites using primers CT-F60 and CT-R60. The primers CT-F61 and CT-R61 were used for site-directed mutagenesis to remove 32 residues from the C-terminus of the enzyme to give pET15b-*MdGAD3ΔC*.

Sequences encoding the His-tag and linker were deleted from the pET15b-*AtGAD1*, -*MdGAD1*, and –*MdGAD2* constructs via site-directed mutagenesis using the primers CT-F67B and CT-R67B, CT-F68 and CT-R68, and CT-F69 and CT-R69, respectively.

### Expression of apple GADs in various organs

RNA was isolated from 1 g of liquid N_2_-ground mature fruit that had been stored at 0°C under 5 kPa CO_2_ and 2 kPa O_2_ for 8 weeks post-harvest or liquid N_2_-ground fully expanded leaves essentially as described previously [14]. Tree branches were also collected in late winter from Simcoe, ON, and the cut ends were submerged in water and the containers placed into a growth chamber (22°C 16 h day/18°C 8 h night) for 10 days to induce flowering and leaf formation. Open flowers and then fully expanded leaves were harvested and stored at -80°C; all tissues were harvested from three separate branches.

Ribonucleic acid (RNA) integrity and quality was verified by formaldehyde gel electrophoresis and then the RNA was treated with DNase I using the TURBO DNA-free kit (Applied Biosystems, Austin, TX) according to the manufacturer’s instructions. One microgram total RNA was used for first-strand cDNA synthesis with Oligo(dT)_20_ and Superscript III (Invitrogen, Carlsbad, CA) at 50°C according to the manufacturer’s protocol. Primers used for quantitative real-time PCR (qPCR) were designed using Primer Express 3 software (Applied Biosystem) with 60°C melting temperature, 40% to 60% GC content, and 50 to 85 bp amplicon size range (Additional file 1: Table S2). Quantitative PCR was performed with an iQ5 Multicolor Real-Time PCR Detection system (BioRad Laboratories, Hercules, CA, USA), using SYBR Green supermix (BioRad Laboratories) to quantify cDNA synthesis. The final concentration of primers was adjusted to 0.2 μM, and the thermal profile of the qPCR reactions was 95°C for 2 min and 40 cycles of 95°C for 10 s, 55°C for 30 s, and 72°C for 15 s. Results of the qPCR were analyzed using the iQ5 2.1 optical system software. Relative expression and data analysis were determined using the 2^-ΔCt^ method [26] and Elongation factor 1-alpha (NCBI GenBank ID MD0000294265) as the housekeeping gene. Two technical replicates were conducted for each biological replicate, and the average ± SE of three biological replicates was determined for each time point, which were arranged randomly in the controlled environment chamber.

### Heterologous expression and purification of recombinant enzymes

All GAD constructs were expressed in *Escherichia coli* BL21 (DE3) cells as described by Gut *et al.*[9] with minor modifications. Briefly, cultures were induced with 0.4 mM isopropyl-β-D-thiogalactopyranoside at 20°C for 16 h. Harvested cells were resuspended in lysis buffer containing 50 mM 4-(2-hydroxyethyl)-1-piperazineethanesulfonic acid (HEPES) pH 7.2, 150 mM NaCl, 1 mM DTT, 0.1 mM PLP, 1 mM PMSF, and SigmaFast protease inhibitor tablets (Sigma). Resuspended cells were lysed via three 30 s pulses at 80% power using a Sonic Dismembrator (Model FB-120, Fisher Scientific) and then centrifuged. The supernatant was passed through a 0.45 μM filter and CaCl_2_ was added to a final concentration of 10 mM prior to loading onto a column packed with Calmodulin Sepharose 4B (GE Healthcare, USA), which was pre-equilibrated with lysis buffer containing 2 mM CaCl_2_. Columns were washed with 20 bed volumes of lysis buffer containing 2 mM CaCl_2_ and proteins were eluted with lysis buffer containing 2 mM ethylene glycol-bis(2-aminoethylether)-N,N,N’,N’-tetraacetic acid. From 2 to 10 mg of purified recombinant GAD was obtained per liter of cell culture.

Several constructs were prepared with 6-His tags and the recombinant proteins were purified via Ni^2+^-resin. Cell culture and harvest were performed as reported above. Briefly, harvested cells were resuspended in lysis buffer (50 mM NaH_2_PO_4_, 300 mM NaCl, 10 mM imidazole, 1 mM PMSF, and SigmaFast protease inhibitor tablets, pH 8) and then lysed. Then lysate was centrifuged and the supernatant was filtered and loaded onto a column containing 0.25 mL His-Select Nickel Affinity Gel (Sigma-Aldrich, USA), which was pre-equilibrated with lysis buffer. The column was caped and the resin resuspended by inversion and then allowed to settle at 4°C for 5 min before draining the lysate from the column. The column was washed with 20 bed volumes each of lysis buffer, followed by wash buffer (50 mM NaH_2_PO_4_, 300 mM NaCl and 20 mM imidazole, 1 mM PMSF, pH 8). Protein was eluted in eight fractions, half a bed volume each, of elution buffer (50 mM NaH_2_PO_4_, 300 mM NaCl, 250 mM imidazole, 1 mM PMSF, pH 8). Fractions with high protein concentrations were pooled and used for further analysis.

The pET5a vector containing petunia *CaM81* was a gift from Dr. Wayne Snedden. Recombinant CaM81 was produced in *E. coli* BL21 (DE3) cells as described previously [27], except that it was produced here without incorporation of ^35^S.

Protein concentrations were determined via the Bio-Rad Protein Assay [28]; all protein samples were stored at -80°C prior to electrophoresis. SDS-PAGE, staining with Coomassie Blue R-250, and western blotting were performed using standard protocols [14].

### Thrombin cleavage of His-tagged GADs

Thrombin cleavage of the 6-His tag on several recombinant GADs was performed with restriction grade thrombin (1U/μl, Novagen, USA). Briefly, thrombin was diluted fifty times in 50 mM sodium citrate pH 6.5 containing 200 mM NaCl, 0.1% PEG-8000 and 50% glycerol. Eluted GAD protein (10 μg) was cleaved in a 50 μl reaction containing 5 μl 10× reaction buffer, and 1 μl diluted thrombin for 20 min at room temperature. Cleaved proteins were purified and concentrated using 0.5 ml Amicon Ultra Centrifugal Filters (EMD Millipore, USA) with a 50 kDa cutoff as per the manufacturer’s instructions.

### *In vitro* assay of GAD activity

GAD activity of cell-free apple extracts was measured as the production of ^14^CO_2_ from radiolabeled glutamate essentially as described previously [23]. The CaM dependence of GAD activity was determined by adding 0.5 mM CaCl_2_ and 0.2 μM bovine CaM in the absence or presence of 100 μM TFP to the reaction mixture. An overlapping 100 mM buffer system was used (pyridine-HCl at pH 4.5, 5.0 and 5.5, and Bis-Tris HCl at pH 5.5, 6.0, 6.5 and 7.0) to determine the pH response of GAD activity. GAD activity at each pH is expressed as nmol ^14^CO_2_ produced per mg protein per minute, after correction for control assays conducted without extract. Data represent the mean ± SE of three separate apples. GAD activity in all assays was proportional to the amount of added cell-free extract and linear with time.

GAD activity of recombinant GAD was assayed in a final volume of 0.5 mL containing 100 mM Bis-Tris HCl at pH 5.8, 6.8, 6.95, 7.1 or 7.25 in the presence of 10% (v/v) glycerol, 1 mM DTT, 0.1 mM PLP, 0.2 mM PMSF at 30°C for 15 min. Reactions were performed with or without 1 mM CaCl_2_ and 0.2 μM recombinant petunia CaM81. One microgram of recombinant GAD was used in most reactions; the exception was those conducted at pH 6.95, 7.1, and 7.25 without Ca^2+^/CaM, which contained 2 μg. All reactions were initiated by adding glutamate to a final concentration of 1 mM and were terminated by adding sulfosalicylic acid to a final concentration of 31.1 mg ml^-1^. The pH was adjusted to 7.0 with 4 M NaOH. Reactions were passed through a 0.45 μm filter and diluted 20× prior to analysis of GABA and glutamate by reverse-phase high performance liquid chromatography after automatic derivatization with *o*-phthalaldehyde as described previously [29]. The mean of three technical replicates is presented for each experimental treatment; all experiments were conducted at least twice.

### Assay of spectral properties

Spectral properties were determined as reported by Gut *et al.*[9].

## Abbreviations

Bis-Tris–HCl: Bis(2-hydroxyethyl)amino-tris(hydroxymethyl)methane; CaM: Calmodulin; CaMBD: Calmodulin binding domain; cDNA: Complementary DNA; DNA: Deoxyribonucleic acid; DTT: Dithiothreitol; GABA: γ-aminobutyrate; GAD: Glutamate decarboxylase; HEPES: 4-(2-hydroxyethyl)-1-piperazineethanesulfonic acid; 6-His: Hexahistidine; ORF: Open reading frame; PLP: Pyridoxal 5′-phosphate; PMSF: Phenylmethanesulfonyl fluoride; PVPP: Polyvinylpolypyrrolidone; qPCR: Quantitative polymerase chain reaction; RNA: Ribonucleic acid; SDS-PAGE: Sodium dodecyl sulphate polyacrylamide gel electrophoresis; TFP: Trifluoperazine; UTR: Untranslated region.

## Authors’ contributions

BJS and GGB conceived the study, and CPT, AZ and JL performed the experiments and carried out the analysis. All authors contributed to design of experiments, interpretation of data and writing the manuscript, and read and approved the final manuscript.

## Supplementary Material

Additional file 1: Table S1 Synthetic oligonucleotides used in this study. **Table S2.** Primers used for qPCR. **Figure S1.** Multiple sequence alignment of selected plant GADs. Click here for file

Additional file 2: Figure S2 SDS-PAGE analysis of expression and purification of various recombinant GAD proteins characterized in Figure 3A-C. Click here for file
