# Supplementary material for: Calmodulin-dependent and calmodulin-independent glutamate decarboxylases in apple fruit
Source: BMC Plant Biol. 2013 Sep 28;13:144. doi: 10.1186/1471-2229-13-144 (PMC3849887; doi:10.1186/1471-2229-13-144)
Supplement: Additional file 1: Table S1 — Synthetic oligonucleotides used in this study. Table S2. Primers used for qPCR. Figure S1. Multiple sequence alignment of selected plant GADs. [file 1471-2229-13-144-S1.docx]

**Additional File 1**

**Identification of calmodulin-dependent and -independent glutamate decarboxylases in apple fruit**

Christopher P. Trobacher, Adel Zarei, Jingyun Liu, Shawn M. Clark, Gale G. Bozzo, and Barry J. Shelp^*^

Department of Plant Agriculture, University of Guelph, 50 Stone Rd E., Guelph, ON N1G 2W1, Canada

**Table S1**

Synthetic oligonucleotides used in this study.

| Primer name | Sequence (5'-3') | Description |
| --- | --- | --- |
| GAD1-FP | TAGCCATATGATGGTGATCTCAACGACTTCC | Amplifying *MdGAD1* ORF |
| GAD1-RP | TAGCGGATCCCTAGCATGCGCCTCTTTTCTTC | See above |
| CT-F32 | ATGGTACTTAAGAGCACTATCCCG | Amplifying *MDP0000284588* with GAD1-RP |
| GAD1R | GAAGTGCTTATGAACATAAAATTGGTAC | Amplifying *MdGAD1* with portions of the 5’ and 3’ UTRs with GAD1-FP |
| CT-F33 | ATGGCTCTCTCAAGGACTGCGTC | Amplifying *MDP0000587459* ORF |
| CT-R33 | CTAACAAACAACATTCATCTTCTGCTTCCTG | See above |
| CT-F37 | CGCTTCGGATGGATCGTTCCGGCATACAC | 3’RACE for *MDP0000587459* (*MdGAD2*) |
| CT-F38 | GCGCAACACATCACCGTGCTACGTGTTG | Nested 3’RACE for *MDP0000587459* (*MdGAD2*) |
| CT-R37 | GGCTCCATCCATGTCGTCACAAACGAAGCC | 5’RACE for *MDP0000587459* (*MdGAD2*) |
| CT-R38 | CCTAGGGTTCCCATCCAACATCAGCTCATCG | Nested 5’RACE for *MDP0000587459* (*MdGAD2*) |
| CT-F39 | GGGCCATATGGCTCTCTCAAGGACTGC | Amplifying *MdGAD2* with 5’ *Nde*I and 3’ *BamH*I sites |
| CT-R39 | CGGATCCTCAACAAACAACATTCATCTTCTGC | See above |
| CT-F34 | ATGGGGCTCTCGAAAACATTCTCAGAG | Amplifying *MDP0000307719* (*MdGAD3*) |
| CT-R34 | CTAGTAGCATTTTGCACCGTCCGTCATC | See above |
| CT-F44 | CTTACAATTAGCTAGCCAGTTTAATTGTGTGATATCTGC | Amplifying GAD3 with 5’ and 3’ UTRs |
| CT-R44 | CCTACCACCGATCTAATAAACTCGATCTCCAACAAC | See above |
| aGL | tagccatatgATGGTGATCTCAACGACTTCC | Amplifying *MdGAD1* with 5’ *Nde*I and 3’ *BamH*I sites |
| aGR | tagcggatccCTAGCATGCGCCTCTTTTCTTC | See above |
| CT-F60 | ACTCGAGATGGGGCTCTCGAAAACATTCTC | Amplifying *MdGAD3* with *Xho*I sites |
| CT-R60 | TCTCGAGCTAGTAGCATTTTGCACCGTC | See above |
| CT-F61 | GAGTTGCCACCAGCTGCTGCTTAGCTCGAGGATCCGGCTGC | Site-directed mutagenesis to remove 32 aa from the C-terminus of pET15b-*MdGAD3* |
| CT-R61 | GCAGCCGGATCCTCGAGCTAAGCAGCAGCTGGTGGCAACTC | See above |
| CT-F67B | CTTTAAGAAGGAGATATACCATGGTGCTCTCCCACGCCG | Site-directed mutagenesis to remove His tag from pET15b-*AtGAD1* |
| CT-R67B | CGGCGTGGGAGAGCACCATGGTATATCTCCTTCTTAAAG | See above |
| CT-F68 | CTTTAAGAAGGAGATATACCATGGTGATCTCAACGACTTCC | Site-directed mutagenesis to remove His tag from pET15b-*MdGAD1* |
| CT-R68 | GGAAGTCGTTGAGATCACCATGGTATATCTCCTTCTTAAAG | See above |
| CT-F69 | CTTTAAGAAGGAGATATACCATGGCTCTCTCAAGGACTGC | Site-directed mutagenesis to remove His tag from pET15b-*MdGAD2* |
| CT-R69 | GCAGTCCTTGAGAGAGCCATGGTATATCTCCTTCTTAAAG | See above |
| AtGAD1 F | GCTCTAGACATATGGTGCTCTCCCAC | Amplifying *AtGAD1* and *AtGAD1ΔCaMBD* with 5’ *Nde*I and 3’ *BamH*I sites |
| AtGAD1 R | CGGGATCCTTAGCAGATACCACTCG | Amplifying *AtGAD1* with 5’ *Nde*I and 3’ *BamH*I sites |
| AtGAD1ΔCaMBD R | CGGGATCCTTACATCAAGTTATCTCTGTTAG | Amplifying *AtGAD1ΔCaMBD* with 5’ *Nde*I and 3’ *BamH*I sites |

**Table S2**

Primers used for qPCR

| Gene name (Locus) | Forward primer (5'-3') | Reverse primer (5'-3') |
| --- | --- | --- |
| *Md*GAD1 (KC812242) | CAGCCAATGCGGAACATGTA | CCGGCTGAAATCCTCCCTAA |
| *Md*GAD2 (KC812243) | AGTAGTTGATGCCGGCTGCTA | CATACTCAGGAGCCCCTTTT |
| *Md*GAd3 (KC812242) | CGGTGGGACAGACACAGAGA | CACTCCGACTAGTAGCATTTTGCA |
| *Md*EF-1 (MD0000294265) | CTCCCACATTGCCGTCAAG | GCCAGATCGCCTGTCGAT |

------------------

*Ph*GAD 1 MVLSKTVSQS----DVSIHSTFASRYVRTSLPRFKMPDNSIPKEAAYQIINDELMLDGNPRLNLASFVTTWMEPECDKLM
*At*GAD1 1 MVLSHAVSES----DVSVHSTFASRYVRTSLPRFKMPENSIPKEAAYQIINDELMLDGNPRLNLASFVTTWMEPECDKLI
*Os*GAD1 1 MVVSVAATDSDTAQPVQYSTFFASRYVRDPLPRFRMPEQSIPREAAYQIINDELMLDGNPRLNLASFVTTWMEPECDKLI
*Os*GAD2 1 MVLTHVEAVE--EGSEAAAAVFASRYVQDPVPRYELGERSISKDAAYQIVHDELLLDSSPRLNLASFVTTWMEPECDRLI
*Md*GAD1 1 MVISTTSAE---GRGEQVNCTFASRYVRNVLPKFQMPETSMPKDSAYQIINDELMLDGNPRLNLASFVTTWMEPECDRLM
*Md*GAD2 1 MALSRTASES----DVSVHSTFASRYVRTSLPRFKMAENSIPKEAAYQIINDELMLDGNPRLNLASFVTTWMEPECDKLM
*Md*GAD3 1 MGLSKTFSES----DVSLHSTTS--YILTSPAKYKMPENSMPEDMAFQMIDDELRLDADPRLNLASFVTTSMEEKAKRLI

 --------------------------------------------------------------------------------
*Ph*GAD 77 MDSINKNYVDMDEYPVTTELQNRCVNMIAHLFNAPLEDGETAVGVGTVGSSEAIMLAGLAFKRKWQNKMKAQGKPCDKPN
*At*GAD1 77 MSSINKNYVDMDEYPVTTELQNRCVNMIAHLFNAPLEEAETAVGVGTVGSSEAIMLAGLAFKRKWQNKRKAEGKPVDKPN
*Os*GAD1 81 MDSVNKNYVDMDEYPVTTELQNRCVNMIAHLFNAPIKEDETAIGVGTVGSSEAIMLAGLAFKRKWQNKRKEQGKPCDKPN
*Os*GAD2 79 LEAINKNYADMDEYPVTTELQNRCVNIIARLFNAPVGDGEKAVGVGTVGSSEAIMLAGLAFKRRWQNRRKAAGKPHDKPN
*Md*GAD1 78 MASMNKNYVDMDEYPVTTELQNRCVNIIANLFNAPIGDGETAVGVSTVGSSEAMMLAGLAFKRKWQNKRKLEGKPFDKPN
*Md*GAD2 77 MASINKNYVDMDEYPVTTELQNRCVNMIAHLFNAPLGDSEAAIGVGTVGSSEAIMLAGLAFKRKWQNKRRAEGKPVDKPN
*Md*GAD3 75 MESLDKNYVDMDEYPATTDLQNRCVNMIAHLFNAPLKDGEAATGTGTVGSSEAIMLAGLAFKRKWQNKMKAIGKPYDKPN

 --------------------------------------------------------------------------------
*Ph*GAD 157 IVTGANVQVCWEKFARYFEVELKEVKLSEGYYVMDPEKAVEMVDENTICVAAILGSTLNGEFEDVKRLNDLLVEKNKETG
*At*GAD1 157 IVTGANVQVCWEKFARYFEVELKEVKLSEGYYVMDPQQAVDMVDENTICVAAILGSTLNGEFEDVKLLNDLLVEKNKETG
*Os*GAD1 161 IVTGANVQVCWEKFARYFEVELKEVKLSEGYYVMDPVKAVEMVDENTICVAAILGSTLTGEFEDVKLLNNLLTEKNKETG
*Os*GAD2 159 IVTGANVQVCWEKFARYFEVELKEVKLTEGCYVMDPVKAVDMVDENTICVAAILGSTLTGEFEDVRRLNDLLAAKNKRTG
*Md*GAD1 158 MVTGANVQVCWEKFARYFEVELKEVKLSEGYYVMDPAKAVEMVDENTICVAAILGSTLTGEFEDVKLLHDLLVEKNKQTG
*Md*GAD2 157 IVTGANVQVCWEKFARYFEVELKEVKLRDGYYVMDPEKAVEMVDENTICVAAILGSTLNGEFEDVKLLNDLLIEKNKETG
*Md*GAD3 155 IVTGANVQVCWEKFARYFEVELKEVKVREDYYVMDPVKAVEMVDENTICVAAILGSTYNGEFEDVKLLNDLLMEKNKQTG

 ----------------------------------------*---------------------------------------
*Ph*GAD 237 WDTPIHVDAASGGFIAPFIYPELEWDFRLPLVKSINVSGHKYGLVYAGIGWVVWRNKDDLPDELIFHINYLGADQPTFTL
*At*GAD1 237 WDTPIHVDAASGGFIAPFLYPELEWDFRLPLVKSINVSGHKYGLVYAGIGWVIWRNKEDLPEELIFHINYLGADQPTFTL
*Os*GAD1 241 WDVPIHVDAASGGFIAPFLYPELEWDFRLPLVKSINVSGHKYGLVYPGVGWVIWRSKEDLPEELIFHINYLGTDQPTFTL
*Os*GAD2 239 WDTPIHVDAASGGFIAPFIYPELEWDFRLPLVKSINVSGHKYGLVYAGVGWVIWRNKEDLPEELIFHINYLGADQPTFTL
*Md*GAD1 238 WDTPIHVDAASGGFIAPFLYPELEWDFRLPLVKSINVSGHKYGLVYAGVGWVVWRSKEDLPDELIFHINYLGSDQPTFTL
*Md*GAD2 237 WDTTIHVDAASGGFIAPFLYPELEWDFRLPLVKSINVSGHKYGLVYAGIGWVIWRNKEDLPEELIFHINYLGADQPTFTL
*Md*GAD3 235 WDTPIHVDAASGGFIAPFLYPDLEWDFRLPLVKSINASGHKYGLVYAGIGWIVWRSKQDLPEDLIFHINYLGADQPTFTL

 --------------------------------------------------------------------------------
*Ph*GAD 317 NFSKGSSQVIAQYYQLIRLGYEGYKNVMENCQENASVLREGLEKTGRFNIISKEIGVPLVAFSLKDNRQHNEFEISETLR
*At*GAD1 317 NFSKGSSQVIAQYYQLIRLGHEGYRNVMENCRENMIVLREGLEKTERFNIVSKDEGVPLVAFSLKDSSCHTEFEISDMLR
*Os*GAD1 321 NFSKGSSQIIAQYYQLIRLGFEGYKNIMQNCMENTAILREGIEATGRFEILSKEAGVPLVAFSLKDSGRYTVFDISEHLR
*Os*GAD2 319 NFSKGSSQIIAQYYQFLRLGFEGYKSVMKNCMESARTLREGLEKTGRFTIISKEEGVPLVAFTFKDGAGAQAFRLSSGLR
*Md*GAD1 318 NFSKGSSQIIAQYYQFIRLGFEGYKNVMENCMENTRMLKQGLEKTGRFKILSKDIGVPLVAFSLKDSSKHTVFEVADSLR
*Md*GAD2 317 NFSKGSSQVIAQYYQLIRLGFEGYRNVMENCRENMVVLKEGLEKTGRFNIVSKDEGVPLVAFSLKDNHRHDEFEISDLLR
*Md*GAD3 315 NFSKGSSQVLAQYYQLIRLGFEGYHEIMENCHYLAMVVKEGLEKTGQFKILSKDIGVPVVAFSLKDRSRYDEFKVSEGLR

 -------------------------------------------
*Ph*GAD 397 RFGWIVPAYTMPPNAQHITVLRVVIREDFSRTLAERLVRDIEKVLHELDTLP----ARVNAKLAVAEEQAAANGSEVHK-
*At*GAD1 397 RYGWIVPAYTMPPNAQHITVLRVVIREDFSRTLAERLVIDIEKVMRELDELP----SRVIHKISLGQEKSESNSDNLMVT
*Os*GAD1 401 RFGWIVPAYTMPANAEHVAVLRVVIREDFSRSLAERLVSDIVKILHELDAHS----AQVLKISSAIAK---QQSGDDGVV
*Os*GAD2 399 RYGWIVPAYTMPAALEHMTVVRVVVREDFGRPLAERFLSHVRMALDEMDLAA----RAPVPRVQLTIELGPARTAGEEAS
*Md*GAD1 398 KFGWTVPAYTMPANAEHVAVLRVVIREDFSRGLAERLISDIDKVMREVDTLP----SQVSSKTAHVTPTVDEVVRDSEVA
*Md*GAD2 397 RFGWIVPAYTMPPDAQHITVLRVVIREDFSRTLAERLVNDIKKVLRELDTLP----SKLSSNVKAADEEGEKPGTTLES-
*Md*GAD3 395 RHQFIVPAYHMPADAKHVALLRVVIRGDFSRTRAEYLLSSINAVLKELDELHPVAAIQENGHHKLSLNGVANGNGELPPA


*Ph*GAD 472 ------KTDSEVQLEMITAWKKFVEEKKKKTNRVC-
*At*GAD1 473 VK----KSDIDKQRDIITGWKKFVADRK-KTSGIC-
*Os*GAD1 474 TK----KSVLETEREIFAYWRDQVKKKQ---TGIC-
*Os*GAD2 475 IR------VVKSEAVPVRKSVPLVAGKT---KGVC-
*Md*GAD1 474 VKSTVHKSETEAEQEIVSRWKGIVKKR-----GAC-
*Md*GAD2 472 -K----KSDLEKTREITIVWRKFVMARKQKMNVVC-
*Md*GAD3 475 AAVENCHHKVSANGAAYGNGGTDTENGQMTDGAKCY

**Figure S1.** Multiple sequence alignment of selected plant GADs. Multiple sequence alignment of *Ph*GAD (AAA33710.1), *At*GAD1 (At5g17330), *Os*GADs 1 and 2 (AB056060, LOC_Os08g36320.1; AB056061, LOC_Os04g37500.1), and *Md*GADs 1-3 (KC812242, KC812243, KC812244) was conducted using ClustalW [16]; identical residues are shown with a black background, and similar residues are shown with a grey background. All enzymes were identified as belonging to the aspartate aminotransferase superfamily (fold type I) of PLP-dependent enzymes by the NCBI CD-Search tool [36]. All enzymes shown, except *Md*GAD3, possess the DOPA decarboxylase family domain, denoted by the dashed line above the sequences. The DOPA decarboxylase family contains DOPA/tyrosine decarboxylases (DDCs), histidine decarboxylases (HDCs), and glutamate decarboxylases (GADs). Within the DDC domain PLP forms an internal aldimine bond (Schiff base linkage) with the catalytic residue lysine marked with an asterisk.
