# Supplementary material for: Calmodulin-dependent and calmodulin-independent glutamate decarboxylases in apple fruit
Source: BMC Plant Biol. 2013 Sep 28;13:144. doi: 10.1186/1471-2229-13-144 (PMC3849887; doi:10.1186/1471-2229-13-144)
Supplement: Additional file 2: Figure S2 — SDS-PAGE analysis of expression and purification of various recombinant GAD proteins characterized in Figure 3A-C. [file 1471-2229-13-144-S2.pptx]

## Slide 1
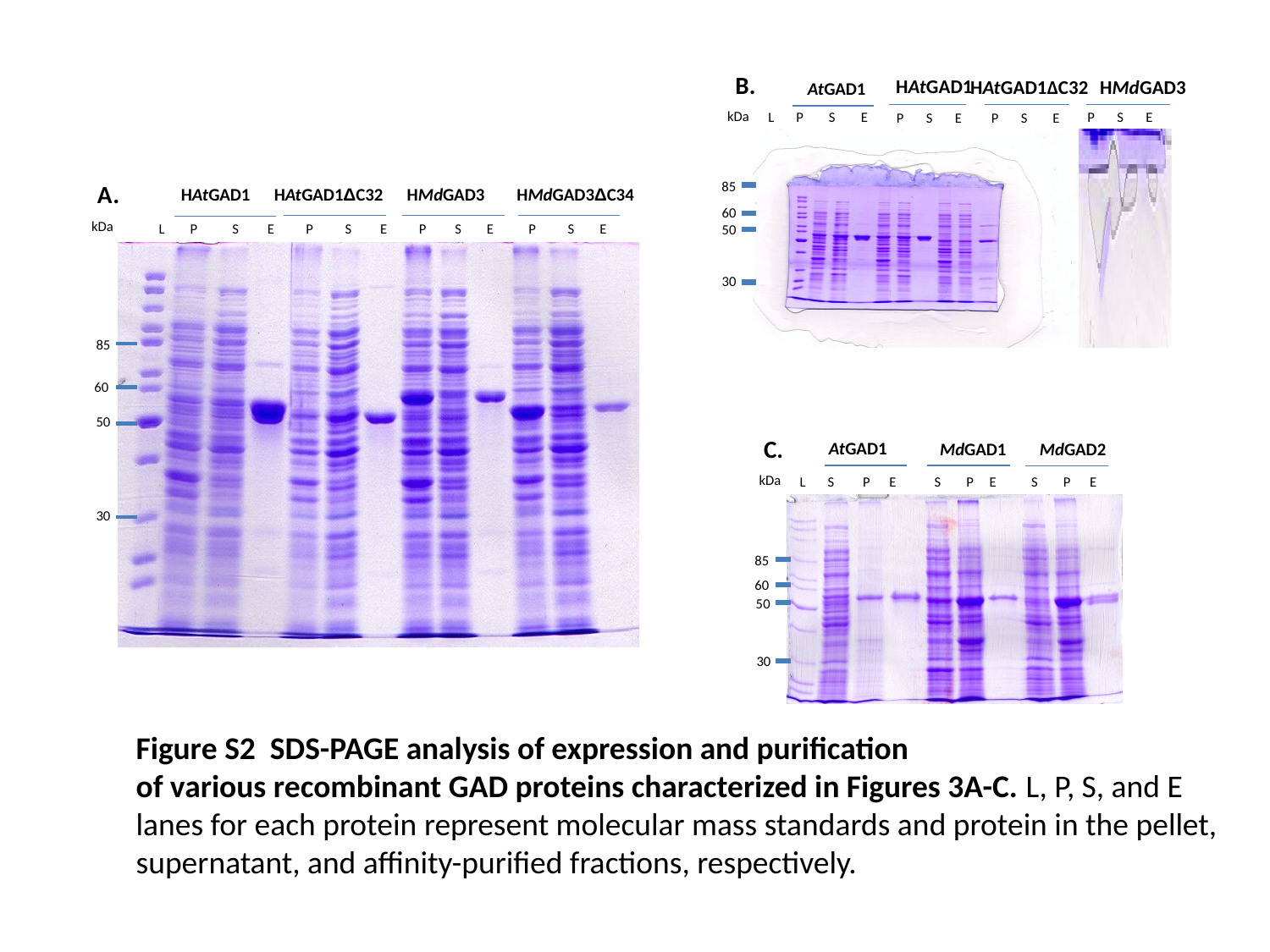

B.
HAtGAD1
 HAtGAD1∆C32
HMdGAD3
AtGAD1
 P S E
 P S E
 P S E
kDa
 L P S E
85
60
50
30
A.
 HAtGAD1 HAtGAD1ΔC32 HMdGAD3 HMdGAD3ΔC34
kDa
L P S E P S E P S E P S E
85
60
50
30
C.
AtGAD1
MdGAD2
MdGAD1
kDa
L S P E S P E S P E
85
60
50
30
Figure S2 SDS-PAGE analysis of expression and purification
of various recombinant GAD proteins characterized in Figures 3A-C. L, P, S, and E
lanes for each protein represent molecular mass standards and protein in the pellet,
supernatant, and affinity-purified fractions, respectively.
